# Recruitment of dlPFC during dietary self-regulation predicts the transience of regulatory effects

**DOI:** 10.1093/scan/nsab088

**Published:** 2021-07-31

**Authors:** Daniel J Wilson, Azadeh HajiHosseini, Cendri A Hutcherson

**Affiliations:** Department of Psychology, University of Toronto, Toronto, ON Canada; Department of Psychology, University of Toronto, Toronto, ON Canada; Department of Psychology, University of Toronto, Toronto, ON Canada; Department of Marketing, Rotman School of Management, University of Toronto, Toronto, ON Canada

**Keywords:** decision-making, dietary choice, cognitive regulation, ventromedial prefrontal cortex, dorsolateral prefrontal cortex

## Abstract

Recent work on the cognitive regulation of dietary decision-making suggests that regulation can alter both the choices that people make in the moment and longer-lasting preferences. However, it is unclear what mechanisms lead to temporary or lingering changes. To address this question, we used fMRI during a task employing the cognitive regulation of food choice and assessed changes in food preference from baseline to post-regulation. We found evidence that regulation may result in a temporary reconfiguration of the neural drivers of choice, de-emphasizing goal-inconsistent value-related computations in the ventromedial prefrontal cortex and resulting in more goal-consistent changes in value-related computations in the dorsolateral prefrontal cortex (dlPFC). Moreover, we find that the extent to which the dlPFC was recruited to represent different regulatory goals during the moment of choice negatively predicted the extent to which those regulatory goals produced lingering changes in preference. Our results suggest that the recruitment of the dlPFC in the service of regulation may have a downside: it is effective at changing behavior in the moment, but its effects on preferences are transient.

## Introduction

One of the perennial laments of dieters the world over centers around the difficulty of maintaining healthy eating practices. All too often, we set a goal to eat healthier or to simply eat less and succeed for a few days or weeks only to have our core love of sugars and fats rear up at an inopportune moment. Recent research has pointed to the potential utility of value-based cognitive self-regulation—the use of memory, attention and executive control to alter the value we place on food—as a potential solution to this problem (Hare *et al.*, 2011). This work suggests not only that cognitive self-regulation can be used to change the momentary values assigned to different foods but also that regulatory effects can persist beyond the moment of active regulatory focus, leading to lasting changes in preference. More remarkably, these changes appear not to require active regulatory effort to maintain (Boswell *et al.*, 2018; see also Denny *et al.*, 2015 for a similar conclusion in the domain of emotion regulation). But this finding raises something of a puzzle: if self-regulatory efforts can alter not only momentary but also lasting preferences, why don’t more people learn to ‘dislike’ cookies and ice cream? What determines whether self-regulatory efforts produce only transient rather than persistent changes in behavior? Here, we suggest that the answer may lie partly in the extent to which distinct neural mechanisms are recruited to accomplish a self-regulatory goal.

Prior research on the neural implementation of cognitive self-regulation in the domain of dietary choice consistently implicates the ventromedial prefrontal cortex (vmPFC) and the dorsolateral prefrontal cortex (dlPFC), although the literature paints a mixed picture regarding the precise role played by each of these regions. Some research suggests that cognitive regulation recruits the dlPFC—an area associated with cognitive control more generally (Brass *et al.*, 2005)—to alter computations in the vmPFC, a region consistently shown to correlate with decision values at the time of choice (Bartra *et al.*, 2013; Clithero and Rangel, 2013). For example, regulation of craving appears to activate portions of the dlPFC and reduce activation in the vmPFC (Kober *et al.*, 2010). Similarly, attempts to consider the healthiness of a food seem to activate the dlPFC, which appears, through functional connectivity, to modulate vmPFC representations of healthiness (Hare *et al.*, 2011). Nevertheless, other research suggests that signals in the vmPFC might sometimes resist alteration by cognitive self-regulation. In these cases, neural patterns of response suggest that the dlPFC might represent a distinct value signal that shows more flexible, goal-consistent effects of regulation. For example, research suggests that attempts to decrease craving sometimes fail to alter vmPFC value signals (Hutcherson *et al.*, 2012; Yokum and Stice, 2013). At the same time, the dlFPC shows reduced activation overall (consistent with the goal to reduce craving) as well as an increased association with explicitly expressed preference (Hutcherson *et al.*, 2012). Other work using multivariate decoding analyses suggests a similar idea. When participants focused on healthy eating, vmPFC value signals showed little change as a function of regulatory condition, while dlPFC signals decoded healthiness more strongly and tastiness less strongly. When participants focused on considerations of tastiness, the opposite was observed (Tusche and Hutcherson, 2018).

It is currently unclear what might account for these discrepant findings. One possibility is that they reflect individual heterogeneity in the strategies or mechanisms used to accomplish regulatory goals. For example, some individuals might first attempt to alter their preferences through processes that target value signals in the vmPFC. If they succeed, no further effort is required. However, in cases where initial attempts to regulate fail to change vmPFC responding, individuals might resort to more effortful processes involving modulation of signals within the dlPFC. To the extent that such efforts are successful, it might be the dlPFC, rather than vmPFC, that ultimately guides the behavioral response. In this view, regulation might target both the vmPFC and the dlPFC, and both areas might be related to regulatory success, depending on the circumstances. Such a view is consistent with the evidence that gray matter in both the vmPFC and the dlPFC positively predicts individual differences in regulatory success across multiple forms of self-control tasks (Schmidt *et al.*, 2018). This view is also consistent with recent research that suggests that both the dlPFC and vmPFC are independently predictive of choice in a sample of dieters focused on losing weight (Cosme *et al.*, 2019).

How might such processes relate to the persistence or transience of self-regulatory efforts? We speculated that computations in the dlPFC might represent a mechanism for temporarily shifting attention toward momentarily goal-relevant outcomes. Such a view is consistent with research suggesting that the dlPFC activates when contexts require a shift in attention toward new stimulus dimensions (Rudorf and Hare, 2014; Leong *et al.*, 2017), as well as evidence that it represents attribute values in a goal-sensitive manner (Tusche and Hutcherson, 2018). However, while this mechanism might contribute to regulatory success in the moment, such effects are unlikely to last much beyond the moment of active regulatory focus since they operate via an additional, effortful route to behavioral control. In contrast, we hypothesized that the successful modulation of value signals in the vmPFC, which has a rich set of interconnections with regions involved in reward learning (e.g. amygdala and ventral striatum: Carmichael and Price, 1995; Haber *et al.*, 2006), might produce more persistent shifts in preference toward regulatory goals. Thus, we predicted that the effects of regulation might have quite different consequences for later preferences, depending on the route by which regulation is accomplished: the recruitment of dlPFC to represent regulatory changes in the absence of alteration of the vmPFC might predict more transient changes limited to the moment of active regulatory focus, while regulation-induced changes in the vmPFC might predict more lingering changes.

Unfortunately, little is known regarding the neural predictors of alterations in preference that linger beyond the moment of active regulation. Related work in the domain of emotion regulation suggests that cognitive reappraisal can produce lingering reductions in amygdala response (Denny *et al.*, 2015) and that this effect might be driven by activity in the dlPFC during the moment of active regulatory focus (Erk *et al.*, 2010). However, no study to date has examined the neural predictors of lingering regulatory effects of cognitive self-regulation on food choice.

We thus aimed to test several related hypotheses about the processes supporting both momentary and lingering dietary change as a result of the cognitive self-regulation of value (i.e. the extent to which the initial liking of a food is altered by regulation in the ∼1 h duration after regulation has occurred). Focusing on regions of the vmPFC and dlPFC showing evidence of value representations at the time of choice, we first asked whether the instructed self-regulation of value produced goal-consistent changes in response in either or both of these regions. To do this, we first focused on two prominent regulatory strategies in the literature that emphasized different goals. In one, participants are asked to focus on a goal to decrease cravings and value for all foods encountered (Hutcherson *et al.*, 2012). In the other, participants are asked to focus on a goal that more specifically emphasizes health considerations (i.e. a goal that decreases the value of unhealthy foods but increases the value of healthy foods: Hare *et al.*, 2011; Bhanji and Beer, 2012; Harris *et al.*, 2013; Tusche and Hutcherson, 2018) This allowed us to test for goal-specific effects of self-regulation on value-based responses in the vmPFC and dlPFC. Second, using a recently developed analytical approach for identifying the unique and independent contribution of different brain regions to behavior (Cosme *et al.*, 2019), we sought to determine whether regulation alters the independent influences of vmPFC and dlPFC on choice behavior. We speculated that, if the vmPFC and dlPFC either represent different types of values or are differentially modulated by regulatory focus, altering their independent contributions to choice behavior might be one route by which to accomplish changes in choice behavior (Hutcherson *et al.*, 2012). Finally, we assessed regulation-induced changes in the liking of foods from baseline to post-regulation, asking whether individual differences in the neural responses of vmPFC and dlPFC during the moment of active regulatory focus predicted either lingering or only momentary effects of regulation.

## Methods

### Participants

Sixty-four healthy, right-handed individuals with normal or corrected-to-normal vision (41 females; mean age, 23.2; range, 18–38) were recruited from the University of Toronto and surrounding community via postering and Facebook groups. Fourteen of these subjects were excluded from analysis: three withdrew before completing the experiment, and 11 had excessive head motion during scanning (see MRI data pre-processing section for exclusion criteria), leaving a total of 50 subjects (34 females; mean age, 23.1; range, 18–38; 26 Asian/Asian Canadian, 10 White/Caucasian, 7 Southeast Asian/Indian, 3 Hispanic/Latino, 1 Southeast Asian/Chinese, 1 African Canadian/Black, 1 Turkish and 1 West Indian). Sample size was selected based on previous successful implementations of the task (Hare *et al.*, 2011; Tusche and Hutcherson, 2018) and was also the largest *N* we could run that was consistent with our budget. Subjects were eligible only if they reported frequent consumption of the types of foods used in the study and had no history of psychiatric or neurological conditions. Subjects were paid $60 for participation with an additional $5 payment for punctuality and a $5 incentive paid to subjects who showed low motion during scanning. All participants gave informed consent and the University of Toronto Research Ethics Board approved all procedures.

### Tasks

#### Overview

Prior to beginning the study, participants fasted for 3 h in order to motivate them to eat foods in the study. In order to assess whether and how neural activation during the cognitive regulation of food value relates to persistent changes in food liking, participants rated a set of 270 images of foods (described below in the Stimuli section) for momentary liking, rating them once before entering the scanner and again after completing all scanner tasks. We defined ‘liking’ for subjects as how much they would like to eat the presented food, in the amount shown on screen, ‘right now’, regardless of other considerations. Then, while in the scanner, participants completed a cognitive regulation task and two executive control tasks (described below). Note that the food images presented to subjects in-scanner during the cognitive regulation task were the same food stimuli that the subjects rated for liking pre- and post-scan, allowing us to assess whether and how regulation targeted at a specific food resulted in lingering changes in liking.

**Fig. 1.**
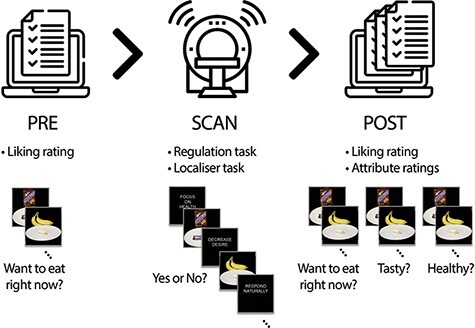
Task design. Participants first completed a set of liking ratings for 270 foods. They then completed a cognitive regulation task in the scanner, in which participants were asked to decide whether or not to eat foods that appeared in one of three instructed regulation conditions (Respond Naturally, Focus on Healthiness, Decrease Desire). Following the completion of the regulation task, participants again rated their preference for all foods, allowing us to assess the effects of regulation on change in liking from baseline to post-task. Finally, they provided attribute ratings for tastiness and healthiness.

After scanning, participants first completed the second set of momentary liking ratings for all food stimuli, and then completed an attribute rating task, in which they made ratings of the subjectively perceived tastiness and healthiness of each food. Note that, while the tastiness of a food is often correlated with momentary liking ratings, these ratings are conceptually distinct, with tastiness referring more to the sensory properties of a food, and liking ratings referring to momentary assessments of preference that can include assessments of tastiness but likely also include other considerations, such as hunger, healthiness, etc. Following the completion of the attribute ratings, a single trial from the cognitive regulation task was randomly drawn and the participant’s choice on that trial determined whether they ate a food or not. If they had indicated Yes or Strong Yes, they ate the food. If they had indicated No or Strong No on that trial, they ate nothing. Finally, participants completed a set of individual differences measures and were debriefed and paid. See Figure 1 for a visualization of the experimental timeline.

#### Stimuli

Prior to conducting the study, we obtained normative ratings of healthiness and tastiness for 165 unique foods, photographed on a black background in multiple different quantities, from a separate sample of 42 individuals. Based on these ratings, we selected 135 different foods, designed to cover the full range of healthiness and tastiness (e.g. candy, chips, fruits and vegetables). Each food was displayed in one of two different quantities (e.g. a small amount of chips and a large amount of chips), presented on a plate or in a container set against a black background (see Figure 1 for examples). This yielded a total of 270 unique food images for use in the tasks described below.

#### Cognitive regulation task

In order to assess how cognitive regulation influences momentary preferences for foods, participants completed the cognitive regulation task inside the scanner. On each trial of the cognitive regulation task, participants were presented with a food image for 4 s, during which time they had to indicate whether they would like to eat the food using a 4-point scale ranging from Strong No to Strong Yes (Figure 1). Importantly, decisions were made in one of three instructed conditions (see Appendix 1 for full instructions): focus on healthiness (HEALTH condition), focus on decreasing all craving (DECREASE condition) and focus on responding naturally (NATURAL condition). On HEALTH trials, subjects were instructed to think about the nutritional and health benefits of the food while making their choice. On DECREASE trials, subjects were told to avoid any craving or emotional response to the food while choosing. On NATURAL trials, subjects were told to allow whatever thoughts and feelings arose while considering the food and to choose based on those feelings. Subjects were instructed to respond honestly regardless of the instruction and were motivated to do so since they were hungry. They knew that one trial would be chosen randomly at the end of the experiment and that they would either receive the trial item (if they had responded ‘Weak Yes’ or ‘Strong Yes’) or receive nothing (if they had responded ‘Weak No’ or ‘Strong No’).

Task blocks began with the presentation of the regulatory instructions (i.e. one of the three regulatory conditions), reminding participants of the mindset to adopt, followed by 10 consecutive trials where subjects were presented with food stimuli and indicated their desire to eat the food. Each scanning run consisted of one block of each instruction (i.e. condition), yielding 30 trials per run. Subjects completed nine runs for a total of 270 trials. In the first run, subjects always began with a block of NATURAL trials. In the remaining runs, the order of the three blocks was randomized. Foods appearing in the regulation task were the same foods that were seen in both the liking rating task and the attribute rating task (described in more detail below).

Importantly, both food quantities of a specific food always appeared in the same condition. Thus, each condition consisted of 45 specific foods presented in two quantities (i.e. 90 food pictures in total). This also meant that each food was only present in a single regulatory condition. The association of specific foods with specific goal conditions allowed us to examine how regulation might persistently alter responses to foods, both generally, across all foods, as well as specifically, as a function of the food appearing in a particular condition. Foods were assigned to each condition for each subject such that baseline liking ratings were matched across conditions (see Liking rating task section below).

#### Executive control tasks

To localize brain areas associated with specific aspects of executive function, subjects also completed two localizer tasks in the scanner following the food choice trials. The first was a go/no-go (GNG) task to test subjects’ ability to resist a prepotent response. The second was an attentional switching task (Rogers and Monsell, 1995). Because these tasks are not the focus of this investigation, we do not describe them further here.

#### Liking rating task

In order to measure lingering changes in liking as a function of cognitive regulation, participants completed two rounds of a liking rating task, outside the scanner, once before and once after the in-scanner cognitive regulation task. During this task, subjects were shown 270 unique food images as described in the Stimuli section above. These were the same food images that subjects saw during the in-scanner cognitive regulation task. Subjects were instructed to indicate how much they would like to eat that food in the amount shown on screen. Subjects completed the task at their own pace using a six-point Likert scale (1, ‘Strongly Dislike’; 6, ‘Strongly Like’). As a reminder, we defined ‘liking’ for subjects as how much they would like to eat the presented food, in the amount shown on screen, ‘right now’, regardless of other considerations. We specified ‘right now’ in an effort to prevent participants from attempting to make their responses consistent across the two rounds of ratings.

#### Attribute rating task

Following the completion of the post-scan liking rating task, and outside the scanner, participants viewed all foods again (270 images of the 135 foods in two different serving sizes) and were asked to rate each food for subjectively perceived tastiness and healthiness without taking into account other considerations. Ratings were made on a Likert Scale from 1 (very untasty/unhealthy) to 6 (very tasty/healthy). All foods were rated for one attribute and then presented a second time and rated for the other attribute. The order in which participants rated the two attributes was randomized.

### Analysis

#### Behavioral analyses

Statistical analyses were conducted in R 3.6.1 (R Core Team, 2019, https://www.R-project.org). To examine how choice behavior differed during cognitive regulation, we computed a regression for each subject and each condition separately with the following fixed effects:

(1) \begin{equation*}Decision\ Valu{e_{Food\ i}} = {\beta _0} + {\beta _1}*Tastines{s_{Food\ i}} + {\beta _2}*Healthines{s_{Food\ i}}.\end{equation*}

To compare across conditions and to correlate individual differences with neural activation, we performed statistical analyses using the subject-level intercept (${\beta _0}$) and slope (${\beta _1},{\beta _2}$) parameters for each variable for each condition.

Making inferences about how liking for foods changes following regulatory effort represents more of a challenge. We thus chose to assess lingering changes in two different ways. First, we computed the raw change over all foods in liking from pre- to post-task (i.e. Post-Task Liking—Pre-Task Liking). However, while this measure can serve as a good overall measure of change, it is less amenable to determining whether specific foods (e.g. foods that are healthier or less health) show specific changes in regulation. This is because foods rated at the negative and positive ends of the scale (i.e. Strong Dislike, Strong Like) are constrained in their direction of movement (i.e. floor and ceiling effects), meaning that pre-task liking may partially determine the degree of change in post-task liking. To account for this issue, we performed a second analysis of change that characterized the effects of regulation on change in post-task liking compared to baseline (${\Delta }Liking$) using the following regression, computed separately for each subject and condition:

(2) \begin{align*}{\Delta }Likin{g_{Food\ i}} =\ &{\beta _0} + {\beta _1}*Tastines{s_{Food\ i}} + {\beta _2}*Healthines{s_{Food\ i}} \cr &+ {\beta _3}*Pre\ Task\ Likin{g_{Food\ i}}.\end{align*}

Examining the raw magnitude of these subject-level coefficients for a given condition permits us to make inferences about how the determinants of food-liking change from baseline. For example, if after controlling for pre-task liking, healthiness predicts a change in liking, then the coefficient on Healthiness should be positive. Comparing the magnitudes of these regression coefficients across conditions allows us to determine whether different forms of regulation result in distinct and persistent changes in liking, beyond the moment of active regulatory focus. Coefficients for the subject-level terms also allow us to examine individual differences and to link them to changes in neural activity during the regulation task.

#### MRI data acquisition

Functional imaging was conducted using a Siemens Prisma 3.0 T MRI scanner, with a gradient strength of 80 mT/m and slew rate of 200 T/m/s. Gradient echo T2*-weighted echoplanar images (EPI) were collected using a 32-channel head coil. To optimize functional sensitivity in the vmPFC, a key region of interest (ROI), we used a tilted acquisition in an oblique orientation of 30° to the anterior commissure–posterior commissure line. Each volume comprised 69 axial slices. A total of 1188 volumes were collected over nine scanning runs (132 volumes/run) during the experiment in a multi-slice interleaved manner to minimize crosstalk between slices. The first two volumes of each run were discarded to allow for scanner equilibration. The imaging parameters were as follows: echo time, 30 ms; field of view, 192 mm; in-plane resolution and slice thickness, 2 mm; repetition time, 2 s. Whole-brain high-resolution T1-weighted structural scans (1 × 1 × 1 mm) were acquired and coregistered with the participant’s mean EPI images. These images were averaged together to permit anatomical localization of the functional activations at the group level.

#### MRI data pre-processing

Pre-processing was performed using the default settings of FMRIPREP software version 1.2.5 (Esteban *et al.*, 2019). In this pre-processing workflow, each T1-weighted (T1w) volume was corrected for intensity non-uniformity using N4BiasFieldCorrection v2.1.0 (Tustison *et al.*, 2010) and then skull-stripped using antsBrainExtraction.sh v2.1.0 (using the OASIS template). Brain surfaces were reconstructed using recon-all from FreeSurfer v6.0.1 (Dale *et al.*, 1999), and the brain mask estimated previously was refined with a custom variation of the method to reconcile ANTs-derived and FreeSurfer-derived segmentations of the cortical gray matter of Mindboggle (Klein *et al.*, 2017). Spatial normalization to the ICBM 152 Nonlinear Asymmetrical template version 2009c (Fonov *et al.*, 2011) was performed through non-linear registration with the antsRegistration tool of ANTs v2.1.0 (Avants *et al*., 2008) using brain-extracted versions of both T1w volume and template. Brain tissue segmentation of cerebrospinal fluid, white matter and gray matter was performed on the brain-extracted T1-weighted image using fast (Zhang *et al.*, 2001).

Functional data were slice time corrected using 3dTshift from AFNI v16.2.07 (Cox, 1996) and motion corrected using mcflirt (Jenkinson *et al.*, 2002). This was followed by co-registration using bbregister (FreeSurfer v6.0.1) to the corresponding T1w image using boundary-based registration (Greve and Fischl, 2009) with 9 degrees of freedom. Motion correcting transformations, BOLD-to-T1w transformation and T1w-to-template (MNI) warp were concatenated and applied in a single step using antsApplyTransforms (ANTs v2.1.0) with Lanczos interpolation. Many internal operations of FMRIPREP use Nilearn (Abraham *et al.*, 2014) principally within the BOLD-processing workflow. For more details of the pipeline, see http://fmriprep.readthedocs.io/en/latest/workflows.html.

Frame-wise displacement (Power *et al.*, 2014) was calculated for each functional run using the implementation in Nipype. After pre-processing, runs that had framewise displacement (FD) measures above threshold (0.2 mm, FMRIPREP) on over 30% of frames were excluded. Runs that had above-threshold FD on 12–30% of frames were included if visual analysis of the carpet plot did not reveal major distortion. Runs with FD below 12% of frames were kept. If subjects had more than three out of the nine total runs removed for excessive FD, they were excluded from the sample. These exclusion criteria resulted in 11 subjects being removed from our analysis due to excessive head motion—in addition to the 3 that withdrew before task completion. Within the 50 subjects kept for analysis, 11 subjects had three runs removed, 8 subjects had two runs removed, 5 subjects had a single run removed and 23 subjects had no runs removed based on the head motion trial exclusion criterion.

#### MRI data analysis

##### GLM 1: neural representations of decision value

Using our pre-processed data, we conducted a general linear model (GLM) with first-order autoregression, as implemented in SPM12, in order to identify regions associated with decision value. This analysis proceeded in three steps. First, we estimated the model separately for each individual. Second, we calculated contrast statistics at the individual level. Third, we computed second-level statistics by carrying out one-sample *t*-tests and correlations on the single-subject contrast coefficients.

The model consisted of six regressors of interest: R1–R3 were indicator functions beginning at the onset of food on each trial and having a duration of the trial’s response time, modeled separately for NATURAL trials (R1), DECREASE trials (R2) and HEALTH trials (R3). Regressors R4–R6 consisted of parametric modulators of each indicator function representing the value of the participant’s preference (from Strong No to Strong Yes) for the specific food on that trial. The model also included six motion parameters (*x*, *y*, *z* translation, as well as pitch, roll and yaw) and session constants as regressors of no interest. To identify voxels in the vmPFC and dlPFC that were consistently associated with value across all three conditions, we calculated a contrast (C1) at the individual level, consisting of the combination of [R4 + R5 + R6]. To examine how activation in vmPFC and dlPFC varied overall as a function of condition, we also calculated subject-level images for NATURAL (R1) HEALTH (R2) and DECREASE (R3) conditions separately.

##### GLM 2: trial-specific beta series responses

Although GLM 1 identified areas whose activation correlates with value, it cannot reveal whether those regions explain ‘independent components’ of the variance in behavioral responses. To do this requires a different approach, in which activation in each region is used to predict behavioral responses after controlling for activation in other regions. For this approach, we used a method developed by Cosme *et al.* (2019), in which trial-by-trial responses from different brain regions are computed and then entered simultaneously into a multiple regression analysis. We thus used GLM 2 to extract these trial-level responses. This GLM consisted of 270 regressors of interest, consisting of a box-car function with an onset at the beginning of each trial and a duration of the subject-specific response time for that trial. All other details were as in GLM 1.

#### Region-of-interest of analyses

Based on GLM 1 described above, we constructed two a priori defined ROIs consisting of 6 mm spheres placed around the peak correlation with decision value (C1) in the vmPFC (centered at *x* = −6, *y* = 24, *z* = 18) and the dlPFC (centered at *x* = 40, *y* = 34, *z* = 18).

Using these two regions, we extracted contrast estimates for (i) overall response in each of the three conditions (GLM 1) using the average across all voxels included in the ROI and (ii) trial-by-trial BOLD response for each subject, averaging the regression weights derived from GLM 2 across all voxels included in the ROI and normalizing these trial-level values within each subject (Cosme *et al.*, 2019).

##### GLM 3

To test whether regulation altered the sensitivity of stimulus-related value signals contained in the vmPFC and dlPFC to tastiness and healthiness, we estimated GLM 3, a model predicting trial-by-trial responses in each region for each subject and each condition separately with the following predictors:

(3) \begin{equation*}RO{I_{Food\ i}} = {\beta _0} + {\beta _1}*Tastines{s_{Food\ i}} + {\beta _2}*Healthines{s_{Food\ i}}\end{equation*}

where ROI_Food_*_i_* represents trial-specific response in either dlPFC or vmPFC. To compare across conditions and to correlate individual differences with neural activation, we performed statistical analyses using the subject-level slope terms for tastiness (${\beta _1}$) and healthiness (${\beta _2}$). Note that, in this analysis, we assume that regions like the vmPFC and dlPFC might serve as regions computing a value signal composed of independent attributes like tastiness and healthiness and that the weighting on these attributes in the neural signal is likely to change as a function of regulatory goals. Although tastiness and healthiness ratings were technically collected ‘after’ the scanning session, they serve as predictors here because we assume that the post-task ratings are quite likely highly correlated with the momentary subjective perceptions of these attributes during the task and serve as proxies for them.

##### GLM 4

To test whether regulation altered the sensitivity of behavior to the value signals contained in the vmPFC and dlPFC (i.e. response-related signals), we estimated a model for each subject and each condition separately with the following predictors of decision value on each trial:

(4) \begin{align*}Decision\ Valu{e_{Food\ i}} =\ &{\beta _0} + {\beta _1}*Tastines{s_{Food\ i}} + {\beta _2}*Healthines{s_{Food\ i}} \cr &+ {\beta _3}*vmPF{C_{Food\ i}} + {\beta _4}*dlPF{C_{Food\ i}}.\end{align*}

To compare across conditions and to correlate individual differences with neural activation, we performed statistical analyses using the subject-level slope terms for the vmPFC (${\beta _3}$) and dlPFC (${\beta _4}$).

## Results

### Behavioral results

#### Momentary effects of regulation

We first sought to verify that participants’ behavior changed in a goal-consistent manner during moments of active cognitive regulation. To do this, we performed one-way repeated-measures ANOVAs with condition (NATURAL, HEALTH and DECREASE) as a fixed effect and three outcome measures as dependent variables: (i) average decision value within each condition, (ii) subject-level slope terms for the influence of tastiness on decision value (see Regression equation (1) in the Methods section for details) and (iii) subject-level slope terms for the influence of healthiness on decision value (see Regression equation (1) in the Methods section for details).

As expected, we found a significant effect of condition on average decision value (*F*_2,98_ = 44.5, *P* < 0.0001). Consistent with regulatory goals, the DECREASE condition resulted in lower decision values on average (*M* = 2.0 ± 0.30 [s.d.]) compared to NATURAL (*M* = 2.3 ± 0.37, paired-*t*_49_ = 7.37, *P* < 0.0001), with the HEALTH condition in between and significantly different from both (*M* = 2.2 ± 0.28, both paired-*t*_49_ >3.73, both *P* < 0.001).

As expected, we also found a significant effect of condition on the influence on choice of both tastiness (*F*_2,98_ = 18.78, *P *< 0.0001) and healthiness (*F*_2,98_ = 59.06, *P* < 0.0001). Consistent with regulatory goals, tastiness influenced choices more strongly during NATURAL trials (mean β* = *0.30 ± 0.15) compared to either HEALTH trials (mean β* = *0.18 ± 0.14, paired-*t*_49_ = 5.25, *P* < 0.0001) or DECREASE trials (mean β* = *0.18 ± 0.16, paired-*t*_49_ = 4.63, *P* < 0.0001), which did not differ significantly from each other (paired-*t*_49_ = 0.13, *P* = 0.81). We also found that healthiness influenced choices more strongly during the HEALTH condition (mean β* = *0.24 ± 0.16) than either the NATURAL condition (mean β* = *0.05 ± 0.11, paired-*t*_49_ = 7.93, *P* < 0.0001) or DECREASE condition (mean β* = *0.053 ± 0.09, paired-t_49_ = 8.63, *P* < 0.0001), which did not differ from each other (paired-*t*_49_ = 0.16, *P* = 0.88).

To examine whether regulation required greater effort, we also compared average RTs across the conditions. As expected, we observed a significant effect of condition on RT (*F*_2,98_ = 13.45, *P *< 0.0001), with responses in both the HEALTH (mean RT = 1404 ms ± 297) and DECREASE (mean RT = 1440 ± 384) conditions taking significantly longer compared to NATURAL (mean RT = 1322 ± 298, both paired-*t*_49_ > 3.91, both *P* < 0.0001).

#### Lingering effects of regulation

We next examined the influence of cognitive regulation on changes in food liking from baseline (ΔLiking) to the post-regulation task (which occurred approximately 1 h after the completion of the regulation task). First, we asked whether regulation (particularly, the DECREASE condition) resulted in a general decrease in liking for all foods. Second, we asked whether regulation (particularly, the HEALTH condition) resulted in an increase in the influence of healthiness (or decrease in the influence of tastiness) for foods appearing in that condition. To test for these lasting effects, we performed one-way ANOVAs with condition (NATURAL, HEALTH and DECREASE) as a fixed effect and subject-level intercept (β_0_) and slope terms for tastiness (β_1_) and healthiness (β_2_) as the dependent measures (see Regression equation (2) in the Methods section for details). As a second way of examining change in overall liking, we also examined the effects of condition on change in raw liking scores from pre- to post-regulation task.

When examining raw change in overall raw liking ratings of all foods, we observed a significant effect of condition (*F*_2,98_ = 4.08, *P* = 0.01), which was driven by a selective overall decrease in liking of foods that appeared in the DECREASE condition relative to both the NATURAL and HEALTH conditions (both *P* < 0.01). Interestingly, when controlling for the effects of tastiness and healthiness and measuring change in liking using the intercept term of the regression, we observed non-significant differences among the conditions in the change in overall liking for foods (*F*_2,98_ = 1.47, *P* = 0.24), although both DECREASE and HEALTH conditions showed non-significant changes in the expected direction (mean DECREASE = 0.37 ± 0.66, mean HEALTH = 0.37 ± 0.91) compared to foods in the NATURAL condition (mean = 0.50 ± 1.06). This suggests that some of the effects we observed might be partially explained by changes in the influence of healthiness and/or tastiness on liking.

Although tastiness predicted greater increases in ΔLiking on average (mean = 0.385 ± 0.155, *F*_1,98_ = 31.4, *P* < 0.0001), we observed no evidence that those changes varied as a function of condition (*F*_2,98_ = 0.12, *P* = 0.89). Note that this lack of difference across conditions in lingering changes in concern for tastiness occurred in the context of strong decreases in the influence of tastiness during on-line regulation. This suggests that some aspects of the changes induced by cognitive regulation may be relatively transient.

By contrast, we observed significant differences between conditions in the extent to which healthiness predicted ΔLiking (*F*_2,98_ = 5.72, *P* < 0.005). As expected, follow-up *t*-tests indicated that healthiness predicted stronger increases in liking specifically for those foods that had appeared in the HEALTH condition (mean = 0.083 ± 0.14) compared both to NATURAL (mean = 0.037 ± 0.12, paired-*t*_49_ = 3.4, *P* = 0.001) and DECREASE trials (mean β_2_ = 0.047 ± 0.13, paired-*t*_49_ = 2.45, *P* = 0.02), which did not differ from each other (paired-*t*_49_ = 0.65, *P = *0.52). Thus, consistent with prior work (Boswell *et al.*, 2018), health-focused regulation led to a significant increase in liking for healthier food targets that endured beyond the moment of active regulatory focus.

### Neural results

#### vmPFC and dlPFC correlate with decision value

**Fig. 2.**
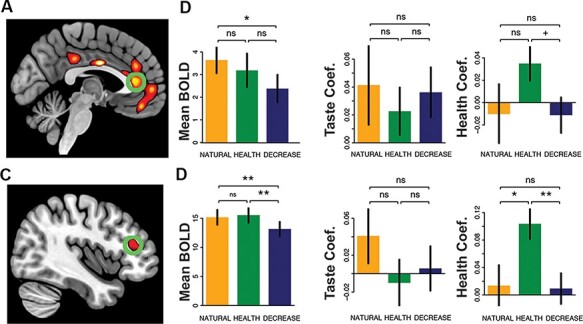
Regulatory effects of stimulus-related responses in the vmPFC and dlPFC. (A) vmPFC was correlated with overall decision value at the time of choice (image thresholded at *P *< 0.00001, uncorrected). The green circle identifies the ROI used to generate the plots to the right. (B) Responses in the vmPFC overall (left) and as a function of food tastiness (middle) and healthiness (right). (C) dlPFC was also correlated with overall decision value at the time of choice. The green circle identifies the ROI used to generate the plots to the right. (D) Responses in the dlPFC overall (left) and as a function of food tastiness (middle) and healthiness (right). Error bars indicate s.e. of the mean. + *P *< 0.06 *P < 0.05 **P < 0.01.

To identify ROIs in the vmPFC and dlPFC, we conducted a GLM in SPM identifying neural correlates of decision value at the time of choice (i.e. response of Strong No to Strong Yes during food choice, GLM 1, Contrast 1) across all three conditions. As expected, this analysis identified both the vmPFC and the right dlPFC (*P* < 0.05, whole-brain corrected, Figure 2A and C, Table 1). We therefore defined 6 mm spherical ROIs around the peak of activation in each region, which we used to extract parameter estimates for further analysis.

**Table 1.** Regions associated with decision value across conditions

	Region	BA	Cluster size	Z score	*x*	*y*	*z*
L	Mid-cingulate cortex	24	18 456	6.18	−2	−4	32
R	Posterior cingulate cortex	31	*	5.73	0	−34	34
L	Medial prefrontal cortex	24	*	5.71	−6	24	18
R	Dorsolateral prefrontal cortex	46	*	5.05	40	34	18
R	Inferior temporal gyrus	20	478	5.14	58	−32	−18
L	Inferior temporal gyrus	20	315	4.39	−54	−34	−14
L	Inferior parietal lobule	40	1208	4.52	−56	−50	56
R	Inferior parietal lobule	40	474	4.38	52	−44	56
L	Precuneus	7	255	3.78	−10	−56	16
L	Cerebellum		207	4.19	−46	−68	−40
R	Cerebellum		166	4.52	46	−70	−42

*Note*: All regions reported are significant at a joint voxel-level *P* < 0.001 and corrected cluster-level *P* < 0.05. *Distinct peak within a larger contiguous region.

#### Regulation-induced change in stimulus-related vmPFC repsonse

We began by asking whether regulation altered responses in the vmPFC in a goal-consistent way. First, we asked whether overall activation in this region changed as a function of regulatory goal using a one-way, repeated-measures ANOVA with condition (NATURAL, HEALTH and DECREASE) as a fixed effect and average response in each condition as the dependent measure. Although we observed a comparatively weak effect of condition on overall response in the vmPFC (*F*_2,98_ = 2.00, *P* = 0.14), follow-up planned comparisons confirmed that activation in the vmPFC was reduced in the DECREASE condition compared to the NATURAL condition (paired-t_49_ 2.33, *P* = 0.02, Figure 2B, left). The comparison between DECREASE and HEALTH trials failed to reach significance (paired-*t*_49_ = 1.16, *P *= 0.25).

Next, we investigated neural sensitivity of the vmPFC to tastiness and healthiness using multiple regression (see Regression equation (3) in the Methods section). Notably, the vmPFC showed a main effect of tastiness across all three conditions (*F*_1,98_ = 6.411, *P *= 0.01), with no effect of condition on this response (Figure 2B, middle, *F*_2,98_ = 0.208, *P *= 0.81). In comparison, we observed no main effect of healthiness in the vmPFC (*F*_1,98_ = 0.154, *P *= 0.70), nor did we observe a significant effect of condition (*F*_2,98_ = 1.75, *P *= 0.18). However, in *posthoc* comparisons, we did observe a marginally significant increase in sensitivity to healthiness in the HEALTH condition compared to the DECREASE condition (*t*_49_ = 1.94, *P *= 0.06, see Figure 2B, right). Thus, responses in this region of the vmPFC showed only relatively modest effects of cognitive regulation.

#### Regulation-induced change in stimulus-related dlPFC response

We next turned to a similar set of analyses in the dlPFC. This analysis suggested that regulation produced a significant and selective decrease in response in this region during DECREASE trials (*F*_2,98_ = 5.34, *P *= 0.006) compared to NATURAL and HEALTH conditions (both paired-*t*_49_ > 2.86, both *P* < 0.006, Figure 2D, left). However, in contrast to the vmPFC, we observed no main effect of tastiness in the dlPFC (*F*_1,98_ = 0.656, *P *= 0.42), nor was there a significant effect of condition on tastiness response (*F*_2,98_ = 0.964, *P *= 0.39, Figure 2D, middle). Finally, we investigated neural sensitivity of the dlPFC to healthiness. This analysis showed a main effect of healthiness in this region (*F*_1,98_ = 6.18, *P *= 0.01) qualified by a significant effect of condition (*F*_2,98_ = 5.624, *P *= 0.005). Follow-up analyses suggested that the dlPFC showed significantly greater responses to healthiness during the HEALTH condition compared to both NATURAL and DECREASE trials (Figure 2D, right, both paired-*t*_49_ > 2.47, both *P *< 0.02).

#### Regulation alters the independent contributions of vmPFC and dlPFC to behavior

The results above suggest two things. First, both the vmPFC and the dlPFC showed some evidence of regulation-consistent changes in the DECREASE and HEALTH conditions, although these effects were somewhat more reliable in the dlPFC compared to the vmPFC. Second, the vmPFC showed a specific, goal-inconsistent sensitivity to tastiness that did not vary by regulatory goal. These results are consistent with past work (Hare *et al.*, 2011; Hutcherson *et al.*, 2012; Tusche and Hutcherson, 2018) and led us to predict that a possible route to regulatory success might be to decrease the influence of the vmPFC on choice behavior and/or to increase the influence of the dlPFC. To test this possibility, for each subject, we conducted regression analyses in which we predicted trial-by-trial decision values simultaneously from trial-by-trial variation in vmPFC and dlPFC response (see Regression equation (4) in the Methods section), controlling for subject-specific tastiness and healthiness ratings. We then conducted repeated-measures one-way ANOVAs with condition as a fixed effect and subject-level influence of dlPFC and vmPFC on choice as the variable to be explained.

**Fig. 3.**
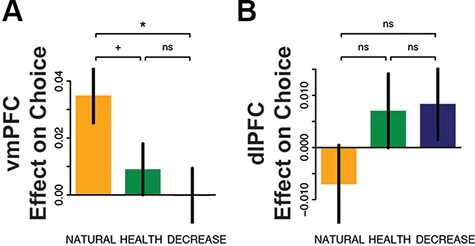
Prediction of decision value responses at the time of choice in vmPFC (A) and dlPFC (B). Error bars indicate s.e. of the mean. + *P *< 0.1 *P < 0.05 **P < 0.01.

We observed a striking alteration in the extent to which vmPFC predicted behavioral responses across the three conditions (Figure 3A). Choice behavior showed a significant difference in sensitivity to signals carried in the vmPFC as a function of condition (*F*_2,98_ = 3.29, *P *= 0.04), driven by a significantly stronger predictive relationship in the NATURAL condition compared to DECREASE (paired-*t*_49_ = 2.31, *P* = 0.03) and (marginally) HEALTH trials (paired-*t*_49_ = 1.78, *P* = 0.08). Results in the dlPFC failed to reach significance (*F*_2,98_ = 2.06, *P *= 0.13, Figure 3B), suggesting that this region did not show a regulation-related change in contribution to choice behavior. Thus, we found evidence of regulation-related shifts in the extent to which the vmPFC, but not the dlPFC, appeared to uniquely predict choice behavior.

#### Neural predictors of the transience of regulatory changes in preference

These results suggest that subjects may have adopted strategies that failed to modulate vmPFC value signals fully in accordance with the regulatory goal. Given the somewhat more robust correspondence of the dlPFC to the regulatory goal (i.e. a drop in activity during DECREASE trials and an increase in sensitivity to healthiness during HEALTH trials), we speculated that this region might help to temporarily compensate in some way for the lack of change in the vmPFC. We thus predicted that dlPFC-related changes in neural response might result in transient, rather than lingering, changes in food preference. To test this idea, we asked whether subjects who showed more or less goal-consistent changes in the vmPFC or dlPFC show systemtic differences in the lingering or transient nature of regulatory effects. We speculated that individuals demonstrating successful, goal-consistent changes in the vmPFC might have more lingering effects of regulation, while individuals demonstrating evidence of goal-consistent responding in the dlPFC might show comparatively more transient effects of regulation.

**Fig. 4.**
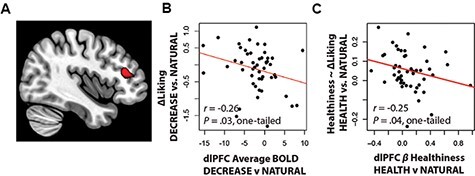
Neural correlates of lingering regulation-induced changes in preferences. (A) dlPFC region correlated with the overall decision value. (B) Goal-consistent decreases in activation in the dlPFC during DECREASE compared to NATURAL trials ‘negatively’ predicted decreases in liking from baseline to post-regulation. (C) Goal-consistent increases in the correlation of the dlPFC with healthiness in HEALTH compared to NATURAL trials ‘negatively’ predicted the extent to which healthiness influenced post-task changes in liking.

Our hypothesis was partially confirmed. We observed a marginal negative correlation between decreases in the dlPFC in the DECREASE condition compared to the NATURAL condition and the extent to which overall liking for foods in the DECREASE condition was suppressed from baseline (Pearson’s *r*_48_ = −0.26, *P* = 0.03, one-tailed, Figure 4A). We also observed a marginal ‘negative’ correlation between dlPFC sensitivity to healthiness in the HEALTH vs. NATURAL conditions and the extent to which healthiness caused an increase in liking for foods from baseline (Pearson’s *r*_48_ = −0.25, *P* = 0.04, one-tailed, Figure 4B). However, no relationship was observed between response in the vmPFC and the durability of regulatory effects (all *P* > 0.39).

#### Neural correlates of regulatory effort

Our results suggest that the dlPFC might be brought on-line temporarily as a way to accomplish regulatory goals. Our theoretical model suggests one further prediction: that this effect might require additional effort to accomplish. Thus, as an exploratory analysis, we asked whether individual differences in RT between the three conditions, which may serve as a proxy for effort, correlated with the extent to which the dlPFC showed goal-consistent change in response. Although we observed no correlation between the increase in RT in the HEALTH condition and response in the dlPFC, we did observe a significant negative correlation between increased RT in the DECREASE compared to NATURAL conditions and the extent to which responses in the dlPFC decreased during this condition (Pearson’s *r*_48_* *= −0.39, *P* = 0.006). Thus, the longer a participant took to choose, the more likely they were to show evidence of a goal-consistent effect of regulation in the dlPFC.

## Discussion

These results advance our understanding of the cognitive self-regulation of dietary choice in several ways. First, they confirm the important roles of both the vmPFC and the dlPFC in value-based decision-making. Second, they support a model in which distinct regulatory goals can produce distinct, goal-consistent changes in stimulus-related response in both the vmPFC and dlPFC, although such effects were more prominent in the dlPFC. Third, they corroborate other accounts (Hutcherson *et al.*, 2012; Tusche and Hutcherson, 2018) suggesting that in contexts where vmPFC shows goal-inconsistent responses (i.e. representing tastiness when goals dictate otherwise), the dlPFC may assume greater responsibility for representing stimulus values in an appropriate way. Finally, they provide evidence about the nature of this compensatory response. It may, in some cases, require extra time to implement and is associated with evidence that the effects of regulation fail to endure beyond the moment of active regulatory focus. These results raise a number of important questions and have several intriguing implications for our understanding of dietary self-regulation in both the short and long term.

One of the key questions implicated by this research concerns the nature of value signals contained in the vmPFC and dlPFC. In one popular view, the vmPFC serves as an integrative hub for attribute-specific value signals received from other areas. In this view, the vmPFC integrates different attributes weighted relative to their current goal values (Basten *et al.*, 2010; Lim *et al.*, 2013; Hutcherson *et al.*, 2015), with the dlPFC acting either as an input to the vmPFC for particular attributes or as a modulator of the weights given by the vmPFC to incoming attribute signals (Hare *et al.*, 2009). In contrast, other work suggests that the vmPFC does not always serve as the final mediator of choice behavior and that the dlPFC might represent an independent input into the process of action selection (Hutcherson *et al.*, 2012).

Our findings are more in line with the latter account. In particular, trial-specific responses in the vmPFC predicted the subject’s behaviorally expressed preference on that trial, over and above other factors like subjectively perceived tastiness and healthiness. However, they did so in different contexts. Signals in the vmPFC contributed to choice more strongly under conditions of naturalistic response but ceased to do so consistently when participants attempted to regulate their decisions. This was true regardless of whether the goal was simply to reduce craving for all foods generally or instead to focus on approaching healthy foods and avoiding unhealthy foods. In contrast, we observed less evidence that the independent contribution of dlPFC responses to behavior changed as a function of regulatory focus.

Our results extend other recent work suggesting that control may in some cases involve an arbitration process over which value signals drive behavior. For example, in the domain of model-free vs. model-based choice, research suggests that regions of the ventrolateral prefrontal cortex (vlPFC) may contribute to this arbitration function between different value systems (Lee *et al.*, 2014). While it remains to be seen whether the vlPFC plays a similar role here, we note that the area of vlPFC identified as the arbitrator in previous work strongly resembles the areas of vlPFC activated in several previous studies of cognitive self-regulation of food choice (e.g. Hutcherson *et al.*, 2012) and emotion regulation (Kohn *et al.*, 2014). Notably, this region was also more active in this study during regulation compared to naturalistic choice (data not shown). Future work will be needed to establish a causal link between the functions of the vlPFC and the determination of the influence of vmPFC signals observed here.

Additional work will also need to examine the precise functional role of the dlPFC region that we observed here. While most models tend to conceptualize the dlPFC as an area that comes online during regulation, we note that the area of the dlPFC that we observed here to correlate with preference, and to decrease in response to instructions to inhibit craving, lies in a location that differentiates it from many other studies of the role of the dlPFC in decision-making. In particular, studies reporting the activation of dlPFC during regulation tend to report coordinates that lie somewhat more dorsally and medially to the area we examined here and tend to report activation on the left compared to the right (Kober *et al.*, 2010; Bhanji and Beer, 2012). Taken together, these results suggest that multiple regions of the dlPFC likely work in concert but that each might perform slightly different computations that together implement effective, although perhaps transient, changes in decision-making behavior.

Several limitations to this work invite a number of open questions and future directions; one of the most important concerns the instructed nature of the regulation conditions examined in this study. While this instructed regulation is commonly deployed in the literature (Kober *et al.*, 2010; Hare *et al.*, 2011; Bhanji and Beer, 2012; Tusche and Hutcherson, 2018), it may not fully resemble the types of spontaneous regulatory strategies that individuals deploy in daily life. Future work will be needed to examine the extent to which our findings might apply to more ecologically valid forms of self-regulation. Our results regarding the lingering effects of regulation also only apply to a relatively short window of time (∼1 h) following the regulation task. While such effects are suggestive, it is important to be cautious in extrapolating from this work to the kinds of regulatory approaches that might change long-term responding to foods on the order of days, months, or even years. Testing the durability of the effect over longer timespans would help make clear whether these strategies could be beneficial beyond the laboratory context. Finally, our focus in this paper centered primarily on two regions, which have been consistently implicated in both valuation and self-control: the vmPFC and the dlPFC. Yet it is likely that other regions, including areas like the ventral striatum, orbitofrontal cortex or hippocampus, might play a role in producing lasting changes in memories and values. Our work points to the fruitfulness of considering the network of changes in the brain that might produce regulatory effects, both in the moment and over longer time periods.

Our results present one answer to why diets may not last: if regulation operates in part by temporarily recruiting the dlPFC to represent stimulus values in a goal-consistent way, but requires effort to maintain, then a loss of focus on the regulatory task may result in a rebound of previous preferences. Indeed, in our study, individuals who showed the strongest goal-consistent stimulus representations in the dlPFC during regulation were also the *least* likely to maintain the effects of regulation on post-regulation preferences. But this result also raises an important question for further research: if recruitment of the dlPFC predicts a failure of regulatory efforts to last beyond the moment of active focus, what mechanisms predict enduring change? In our study, we saw evidence that a focus on healthiness in the moment may result in increases in the value of healthy foods afterward. We had predicted that the changes in the vmPFC might correlate with such effects, but they did not. One possibility is that more sophisticated, multivariate analyses might yield clearer correlates of enduring change. Another possibility is that lasting change might result from connectivity between value regions and regions associated with memory and reward learning (e.g. hippocampus and ventral striatum: Wimmer and Shohamy, 2012; Gerraty *et al.*, 2014). Future work will be needed to test these ideas.

## Supplementary Material

nsab088_Supp

## Appendix 1. Full instructions for all conditions in the Cognitive Regulation Task

In this study, we are interested in understanding people’s ability to modulate their food cravings. There will be three types of trials, where we will ask you to respond in one of the following ways

- Consider whether the food is HEALTHY
- DECREASE your desire for the food
- RESPOND NATURALLY and allow yourself to want the food as much or as little as feels natural

When you see the cue RESPOND NATURALLY, you should choose as naturally as possible. Allow whatever thoughts or feelings arise and simply make your choice.

When you see the cue FOCUS ON HEALTHINESS, you should try to think about whether the food is healthy. Think carefully about the nutritional and health benefits of eating the food. You should continue to look at the food the whole time and focus on the healthiness of the food as you decide whether or not you would like to eat it.

When you see the cue DECREASE DESIRE, you should do whatever you need to decrease your desire for the food. You should continue to look at the food the whole time and try to avoid any craving or emotional response to the food as you decide whether or not you would like to eat the food.

During the choice task, trials will be divided into blocks of 10. Before each block of 10 trials, we will display an instructional cue describing which task you should do for all 10 trials in that block of trials.

The cue will also be shown before each trial at the bottom of the screen as a reminder.

After the instructional cue telling you what to do at the beginning of the trial, the first trial in the block will begin. It is very important that you respond honestly and consider the instructional cue for each trial in the block. You should try to keep the instructed consideration in mind as strongly as possible when making your choices.

However, you should also keep in mind that no matter what type of block you are in, you are ALWAYS FREE TO DECIDE AS YOU PLEASE whether or not to eat a food item. For example, you are free to choose to eat or not eat any food item you want during a health or decrease block if you wish to do so. Similarly, you are free to choose to eat or not eat any food item during a natural block if you prefer.

You may notice that some choices seem very similar. You should always consider your preference for the *exact* food shown, *in the exact amount*, on that trial. However, **only one trial** will be selected to determine what you eat at the end of the study. Whatever you chose on that trial, regardless of the instruction, will be exactly what you receive.

Therefore, your decisions on other trials should not affect what you do on the current trial. On every trial, just choose what you prefer after focusing on the instructions for that trial.

## References

[R1] Abraham A. , Pedregosa F., Eickenberg M., et al. (2014). Machine learning for neuroimaging with scikit-learn. *Frontiers in Neuroinformatics*, 8, 14. 10.3389/fninf.2014.00014 PMC3930868 24600388

[R2] Avants B.B. , Epstein C.L., Grossman M., Gee J.C. (2008). Symmetric diffeomorphic image registration with cross-correlation: evaluating automated labeling of elderly and neurodegenerative brain. *Medical Image Analysis*, 12, 26–41. 17659998 10.1016/j.media.2007.06.004 PMC2276735

[R3] Bartra O. , McGuire J.T., Kable J.W. (2013). The valuation system: a coordinate-based meta-analysis of BOLD fMRI experiments examining neural correlates of subjective value. *NeuroImage*, 76, 412–27. 23507394 10.1016/j.neuroimage.2013.02.063 PMC3756836

[R4] Basten U. , Biele G., Heekeren H.R., Fiebach C.J. (2010). How the brain integrates costs and benefits during decision making. *Proceedings of the National Academy of Sciences*, 107, 21767–72. 10.1073/pnas.0908104107 PMC3003102 21118983

[R5] Bhanji J.P. , Beer J.S. (2012). Taking a different perspective: mindset influences neural regions that represent value and choice. *Social Cognitive and Affective Neuroscience*, 7, 782–93. 21972426 10.1093/scan/nsr062 PMC3475361

[R6] Boswell R.G. , Sun W., Suzuki S., Kober H. (2018). Training in cognitive strategies reduces eating and improves food choice. *Proceedings of the National Academy of Sciences*, 115, E11238–47. 10.1073/pnas.1717092115 PMC6275472 30420496

[R7] Brass M. , Derrfuss J., Forstmann B., von Cramon D.Y. (2005). The role of the inferior frontal junction area in cognitive control. *Trends in Cognitive Sciences*, 9, 314–6. 15927520 10.1016/j.tics.2005.05.001

[R8] Carmichael S.T. , Price J.L. (1995). Limbic connections of the orbital and medial prefrontal cortex in macaque monkeys. *Journal of Comparative Neurology*, 363, 615–41. 8847421 10.1002/cne.903630408

[R9] Clithero J.A. , Rangel A. (2013). Informatic parcellation of the network involved in the computation of subjective value. *Social Cognitive and Affective Neuroscience*, 9, 1289–302. 23887811 10.1093/scan/nst106 PMC4158359

[R10] Cosme D. , Ludwig R.M., Berkman E.T. (2019). Comparing two neurocognitive models of self-control during dietary decisions. *Social Cognitive and Affective Neuroscience*, 14, 957–66. 31593247 10.1093/scan/nsz068 PMC6917023

[R11] Cox R.W. (1996). AFNI: software for analysis and visualization of functional magnetic resonance neuroimages. *Computers and Biomedical Research*, 29, 162–73. 8812068 10.1006/cbmr.1996.0014

[R12] Dale A.M. , Fischl B., Sereno M.I. (1999). Cortical surface-based analysis: I. Segmentation and surface reconstruction. *NeuroImage*, 9, 179–94. 9931268 10.1006/nimg.1998.0395

[R13] Denny B.T. , Inhoff M.C., Zerubavel N., Davachi L., Ochsner K.N. (2015). Getting over it: long-lasting effects of emotion regulation on amygdala response. *Psychological Science*, 26, 1377–88. 26231911 10.1177/0956797615578863 PMC4567486

[R14] Erk S. , Mikschl A., Stier S., et al. (2010). Acute and sustained effects of cognitive emotion regulation in major depression. *Journal of Neuroscience*, 30, 15726–34. 21106812 10.1523/JNEUROSCI.1856-10.2010 PMC6633759

[R15] Esteban O. , Markiewicz C.J., Blair R.W., et al. (2019). fMRIPrep: a robust preprocessing pipeline for functional MRI. *Nature Methods*, 16, 111–6. 30532080 10.1038/s41592-018-0235-4 PMC6319393

[R16] Fonov V. , Evans A.C., Botteron K., Almli C.R., McKinstry R.C., Collins D.L., Brain Development Cooperative, G. (2011). Unbiased average age-appropriate atlases for pediatric studies. *NeuroImage*, 54, 313–27. 20656036 10.1016/j.neuroimage.2010.07.033 PMC2962759

[R17] Gerraty R.T. , Davidow J.Y., Wimmer G.E., Kahn I., Shohamy D. (2014). Transfer of learning relates to intrinsic connectivity between hippocampus, ventromedial prefrontal cortex, and large-scale networks. *The Journal of Neuroscience*, 34, 11297–303. 25143610 10.1523/JNEUROSCI.0185-14.2014 PMC4138340

[R18] Greve D.N. , Fischl B. (2009). Accurate and robust brain image alignment using boundary-based registration. *NeuroImage*, 48, 63–72. 19573611 10.1016/j.neuroimage.2009.06.060 PMC2733527

[R19] Haber S.N. , Kim K.-S., Mailly P., Calzavara R. (2006). Reward-related cortical inputs define a large striatal region in primates that interface with associative cortical connections, providing a substrate for incentive-based learning. *The Journal of Neuroscience*, 26, 8368–76. 16899732 10.1523/JNEUROSCI.0271-06.2006 PMC6673798

[R20] Hare T.A. , Camerer C.F., Rangel A. (2009). Self-control in decision-making involves modulation of the vmPFC valuation system. *Science*, 324, 646–8. 19407204 10.1126/science.1168450

[R21] Hare T.A. , Malmaud J., Rangel A. (2011). Focusing attention on the health aspects of foods changes value signals in vmPFC and improves dietary choice. *The Journal of Neuroscience*, 31, 11077–87. 21795556 10.1523/JNEUROSCI.6383-10.2011 PMC6623079

[R22] Harris A. , Hare T., Rangel A. (2013). Temporally dissociable mechanisms of self-control: early attentional filtering versus late value modulation. *The Journal of Neuroscience*, 33, 18917–31. 24285897 10.1523/JNEUROSCI.5816-12.2013 PMC4018478

[R23] Hutcherson C.A. , Plassmann H., Gross J.J., Rangel A. (2012). Cognitive regulation during decision making shifts behavioral control between ventromedial and dorsolateral prefrontal value systems. *The Journal of Neuroscience*, 32, 13543–54. 23015444 10.1523/JNEUROSCI.6387-11.2012 PMC3689006

[R24] Hutcherson C.A. , Montaser-Kouhsari L., Woodward J., Rangel A. (2015). Emotional and utilitarian appraisals of moral dilemmas are encoded in separate areas and integrated in ventromedial prefrontal cortex. *The Journal of Neuroscience*, 35, 12593–605. 26354924 10.1523/JNEUROSCI.3402-14.2015 PMC4563040

[R25] Jenkinson M. , Bannister P., Brady M., Smith S. (2002). Improved optimization for the robust and accurate linear registration and motion correction of brain images. *NeuroImage*, 17, 825–41. 12377157 10.1016/s1053-8119(02)91132-8

[R26] Klein A. , Ghosh S.S., Bao F.S., et al. (2017). Mindboggling morphometry of human brains. *PLoS Computational Biology*, 13, e1005350. 10.1371/journal.pcbi.1005350 PMC5322885 28231282

[R27] Kober H. , Mende-Siedlecki P., Kross E.F., et al. (2010). Prefrontal–striatal pathway underlies cognitive regulation of craving. *Proceedings of the National Academy of Sciences*, 107, 14811–6. 10.1073/pnas.1007779107 PMC2930456 20679212

[R28] Kohn N. , Eickhoff S.B., Scheller M., Laird A.R., Fox P.T., Habel U. (2014). Neural network of cognitive emotion regulation—an ALE meta-analysis and MACM analysis. *NeuroImage*, 87, 345–55. 24220041 10.1016/j.neuroimage.2013.11.001 PMC4801480

[R29] Lee S.W. , Shimojo S., O’Doherty J.P. (2014). Neural computations underlying arbitration between model-based and model-free learning. *Neuron*, 81, 687–99. 24507199 10.1016/j.neuron.2013.11.028 PMC3968946

[R30] Leong Y.C. , Radulescu A., Daniel R., DeWoskin V., Niv Y. (2017). Dynamic interaction between reinforcement learning and attention in multidimensional environments. *Neuron*, 93, 451–63. 28103483 10.1016/j.neuron.2016.12.040 PMC5287409

[R31] Lim S.L. , O’Doherty J.P., Rangel A. (2013). Stimulus value signals in ventromedial PFC reflect the integration of attribute value signals computed in fusiform gyrus and posterior superior temporal gyrus. *The Journal of Neuroscience*, 33, 8729–41. 23678116 10.1523/JNEUROSCI.4809-12.2013 PMC3865515

[R32] Power J.D. , Mitra A., Laumann T.O., Snyder A.Z., Schlaggar B.L., Petersen S.E. (2014). Methods to detect, characterize, and remove motion artifact in resting state fMRI. *NeuroImage*, 84, 320–41. 23994314 10.1016/j.neuroimage.2013.08.048 PMC3849338

[R0033a] R Core Team (2019). R: A language and environment for statistical computing. R Foundation for Statistical Computing, Vienna, Austria. https://www.R-project.org/.

[R33] Rogers R.D. , Monsell S. (1995). Costs of a predictible switch between simple cognitive tasks. *Journal of Experimental Psychology: General*, 124, 207–31.

[R34] Rudorf S. , Hare T.A. (2014). Interactions between dorsolateral and ventromedial prefrontal cortex underlie context-dependent stimulus valuation in goal-directed choice. *The Journal of Neuroscience*, 34, 15988–96. 25429140 10.1523/JNEUROSCI.3192-14.2014 PMC6608472

[R35] Schmidt L. , Tusche A., Manoharan N., Hutcherson C., Hare T., Plassmann H. (2018). Neuroanatomy of the vmPFC and dlPFC predicts individual differences in cognitive regulation during dietary self-control across regulation strategies. *The Journal of Neuroscience*, 38, 5799–806. 29866743 10.1523/JNEUROSCI.3402-17.2018 PMC6246877

[R36] Tusche A. , Hutcherson C.A. (2018). Cognitive regulation alters social and dietary choice by changing both domain-general and domain-specific attribute representations. *eLife*, 7, e31185. 10.7554/eLife.31185 PMC5973829 29813018

[R37] Tustison N.J. , Avants B.B., Cook P.A., et al. (2010). N4ITK: improved N3 bias correction. *IEEE Transactions on Medical Imaging*, 29, 1310–20. 20378467 10.1109/TMI.2010.2046908 PMC3071855

[R38] Wimmer G.E. , Shohamy D. (2012). Preference by association: how memory mechanisms in the hippocampus bias decisions. *Science*, 338, 270–3. 23066083 10.1126/science.1223252

[R39] Yokum S. , Stice E. (2013). Cognitive regulation of food craving: effects of three cognitive reappraisal strategies on neural response to palatable foods. *International Journal of Obesity*, 37, 1565–70. 23567923 10.1038/ijo.2013.39 PMC3709002

[R40] Zhang Y. , Brady M., Smith S. (2001). Segmentation of brain MR images through a hidden Markov random field model and the expectation-maximization algorithm. *IEEE Transactions on Medical Imaging*, 20, 45–57. 11293691 10.1109/42.906424

