# Supplementary material for: Recruitment of dlPFC during dietary self-regulation predicts the transience of regulatory effects
Source: Soc Cogn Affect Neurosci. 2021 Jul 31;18(1):nsab088. doi: 10.1093/scan/nsab088 (PMC10074768; doi:10.1093/scan/nsab088)
Supplement: nsab088_Supp [file nsab088_supp.zip › scan-20-049-File007.docx]

**Appendix 1. Full instructions for all conditions in the Cognitive Regulation Task**

In this study, we are interested in understanding people’s ability to modulate their food cravings. There will be three types of trials, where we will ask you to respond in one of the following ways:

- Consider whether the food is HEALTHY
- DECREASE your desire for the food
- RESPOND NATURALLY, and allow yourself to want the food as much or as little as feels natural

When you see the cue RESPOND NATURALLY, you should choose as naturally as possible. Allow whatever thoughts or feelings arise, and simply make your choice.

When you see the cue FOCUS ON HEALTHINESS, you should try to think about whether the food is healthy. Think carefully about the nutritional and health benefits of eating the food. You should continue to look at the food the whole time, and focus on the healthiness of the food as you decide whether or not you would like to eat it.

When you see the cue DECREASE DESIRE, you should do whatever you need to decrease your desire for the food. You should continue to look at the food the whole time, and try to avoid any craving or emotional response to the food as you decide whether or not you would like to eat the food.

During the choice task, trials will be divided into blocks of 10. Before each block of 10 trials, we will display an instructional cue describing which task you should do for all 10 trials in that block of trials.

The cue will also be shown before each trial at the bottom of the screen as a reminder.

After the instructional cue telling you what to do at the beginning of the trial, the first trial in the block will begin. It is very important that you respond honestly and consider the instructional cue for each trial in the block. You should try to keep the instructed consideration in mind as strongly as possible when making your choices.

However, you should also keep in mind that no matter what type of block you are in, you are ALWAYS FREE TO DECIDE AS YOU PLEASE whether or not to eat a food item. For example, you are free to choose to eat or not eat any food item you want during a health or decrease block if you wish to do so. Similarly, you are free to choose to eat or not eat any food item during a natural block if you prefer.

You may notice that some choices seem very similar. You should always consider your preference for the *exact* food shown, *in the exact amount*, on that trial. However, **only one trial** will be selected to determine what you eat at the end of the study. Whatever you chose on that trial, regardless of the instruction, will be exactly what you receive.

Therefore your decisions on other trials should not affect what you do on the current trial. On every trial, just choose what you prefer after focusing on the instructions for that trial.
